# Editorial: Physiology in extreme conditions: Adaptations and unexpected reactions, Volume II

**DOI:** 10.3389/fphys.2023.1181010

**Published:** 2023-03-14

**Authors:** Maria G. Trivella, Enrico Capobianco, Antonio L’Abbate

**Affiliations:** ^1^ Consiglio Nazionale delle Ricerche, Institute of Clinical Physiology, Pisa, Italy; ^2^ The Jackson Laboratory, Computational Science, Farmington, CT, United States; ^3^ Scuola Superiore Sant’Anna, Pisa, Italy

**Keywords:** extreme environments, adaptation, underwater, space, altitude, confined environment, heart failure

This Research Topic is an extension of our previous Physiology in extreme conditions (Vol. I), and a first general observation is that Vol. II confirms some of the conclusion reported in Vol. I with reference to two major identified needs:

(a) Enhancing our knowledge and understanding of individual reactions and adaptation mechanisms observed in specific and unusually severe environmental conditions;
(b) Increasing both the availability and the accessibility of data generated in extreme conditions, with the possibility to infer more about both variability and protective capacities among individuals.

A few case studies were presented in the articles of Vol. II covering preclinical models (Desruelle et al.; Siamwala et al.; Bao et al.; Niu et al.) and clinical work, with the latter involving healthy individuals observed in non-physiological environments (Shved et al.) and severely ill children subjected to specific pharmacological treatments (Adorisio et al.) commonly used in adult patients for many years (Packer et al., 1996; Agarwal and venugopalan, 2001).

Then, our curated Research Topic of contributed work includes animal models whose reference scenarios are states of adaptation to unfavourable environments. For instance, the association between a Cecal compound and metabolomic signatures is relevant, which emphasizes individual differences by analysing metabolic compound effects combined with host/microbial communities in diver rats (Desruelle et al., in review).

A first important remark is that well-conducted preclinical models help interpret complex phenomena affecting human beings, like those occurring in extreme conditions. It is worth mentioning that the expectation here is to increase our ability to understand the correct behaviours that may limit or even avoid potentially damaging impacts on health.

A second important remark is that a few key health warnings are identified in the published studies. They include: A) Avoiding decompression illness, B) Stressing the role of antioxidant protection in adapting to height, C) Understanding the effects of space on the structural physiological mechanisms and their angiogenesis- and/or osteogenesis-induced modifications, together with the genetic factors (Siamwala et al.).

In particular, the decompression illness is a well-known threat to the safety of submariners, noting that the maximum depth at which a safe escape is possible is unknown and that targeted studies of maximum safe escape at different depth were carried out in rabbits’ models (Bao et al.).

Significant results emerge from the study on Nanorana parkeri (common names: High Himalaya frog) (Niu et al.) that demonstrated differentiality between high- and low-altitude populations regarding factors such as robust hematologic parameters, less oxidative damage, and stronger antioxidant protection, all contributing to increased survival in high-altitude environments.

A third remark is for clinical trials, which require additional analysis.

The isolation in a confined environment of a small group of “healthy” subjects of mixed gender, defined as “experiment with short-term isolation” (Shved et al.) induces in general adaptation to the stressful environment with different behaviours of extrovert versus introvert subjects, pointing out individual characteristics. Whether living in space could represent a future possibility or not, the basic problem that emerges is knowing the adaptation mechanisms given the induced changes and what countermeasures are appropriate to avoid pathological conditions. In Vol I^o^, a study conducted by Demontis et al. (2017) on the human pathophysiological adaptations to space indicated the need to select well-trained healthy individuals carefully. In Vol II^o^, an additional factor became useful for evaluating astronauts’ skillset, one typically part of the psychological profile (Shved et al.).

Other considerations are needed for the cardiology work on small decompensated paediatric patients (Adorisio et al.). Here the high frequency physiologically present in children (Fleming et al., 2011), also accentuated by the pathological state, is shown to be significantly reduced after pharmacological treatment. This ultimately leads to a reduction of disease severity and/or healing, in line with the beneficial effects of vagus compared to the prevalence of sympathetic in cardiovascular balance (Capilupi et al., 2020). Since the improvement of the left ventricular ejection fraction was enhanced by a major reduction in heart rate (HR), it appears evident that HR could be used as a clinical marker during the treatment of heart failure in children. This evidence could mean a main change and an alternative route in therapy because reducing the need for ventricular assist device implantation as an intermediate procedure before heart transplantation (Di Molfetta et al., 2016; Villa and Morales, 2017).

**FIGURE 1:**
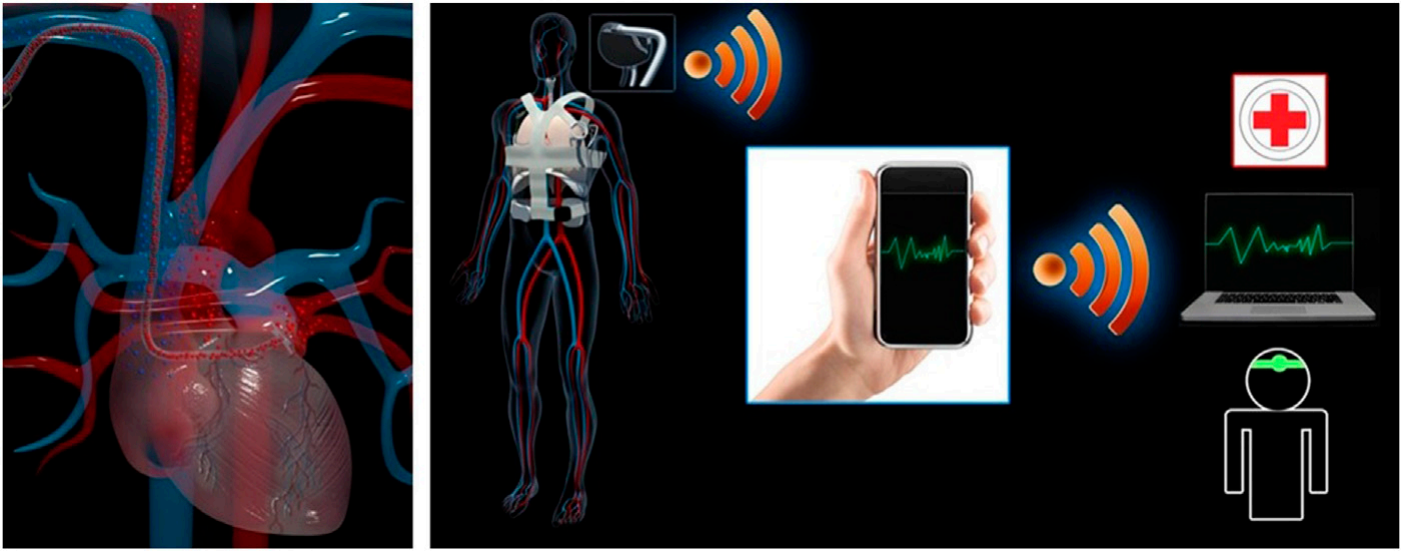
Healthy vs. diseased heart: beyond the therapeutic frontiers, VAD as transient therapeutic tool for heart recovery and/or possible gene therapy. Original author and copyright owner: MG Trivella.

In review, it is important to mention that heart failure of different degrees and the possible health recovery involve understanding adaptation and homeostasis recovery mechanisms. The literature offers examples (i.e., ventricular assistance systems (VAD) solutions as per Simon et al., 2005; Dandel and Hetzer, 2015; Nesta et al., 2021) (Figure 1). Adorisio et al. show that different treatments could help in understanding adaptation. Also, future gene therapies could add valuable information on this Research Topic (Lim, 2019; Giacca, 2020; Kaur and Zangi, 2020).
